# Mutation Analysis in Cultured Cells of Transgenic Rodents

**DOI:** 10.3390/ijms19010262

**Published:** 2018-01-16

**Authors:** Ahmad Besaratinia, Albert Zheng, Steven E. Bates, Stella Tommasi

**Affiliations:** 1 Department of Preventive Medicine, University of Southern California Keck School of Medicine, University of Southern California, M/C 9603, Los Angeles, CA 90033, USA; allegrobertitis@gmail.com (A.Z.); tommasi@med.usc.edu (S.T.); 2 Department of Cancer Biology, Beckman Research Institute of the City of Hope, Duarte, CA 91010, USA; stephi1954@yahoo.com

**Keywords:** assay, *cII* transgene, carcinogen, mouse embryonic fibroblasts, mutation

## Abstract

To comply with guiding principles for the ethical use of animals for experimental research, the field of mutation research has witnessed a shift of interest from large-scale in vivo animal experiments to small-sized in vitro studies. Mutation assays in cultured cells of transgenic rodents constitute, in many ways, viable alternatives to in vivo mutagenicity experiments in the corresponding animals. A variety of transgenic rodent cell culture models and mutation detection systems have been developed for mutagenicity testing of carcinogens. Of these, transgenic Big Blue^®^ (Stratagene Corp., La Jolla, CA, USA, acquired by Agilent Technologies Inc., Santa Clara, CA, USA, BioReliance/Sigma-Aldrich Corp., Darmstadt, Germany) mouse embryonic fibroblasts and the λ Select *cII* Mutation Detection System have been used by many research groups to investigate the mutagenic effects of a wide range of chemical and/or physical carcinogens. Here, we review techniques and principles involved in preparation and culturing of Big Blue^®^ mouse embryonic fibroblasts, treatment in vitro with chemical/physical agent(s) of interest, determination of the *cII* mutant frequency by the λ Select *cII* assay and establishment of the mutation spectrum by DNA sequencing. We describe various approaches for data analysis and interpretation of the results. Furthermore, we highlight representative studies in which the Big Blue^®^ mouse cell culture model and the λ Select *cII* assay have been used for mutagenicity testing of diverse carcinogens. We delineate the advantages of this approach and discuss its limitations, while underscoring auxiliary methods, where applicable.

## 1. Introduction

The contemporary field of mutation research has witnessed a shift of interest from in vivo to in vitro mutagenicity testing to meet the requirements of “reducing” the number of animals used, “refining” procedures to alleviate animal pain, suffering or distress and “replacing” animal use with viable alternatives. These are the “3Rs” known as the guiding principles for ethical use of animals for experimental research [1]. Mutation assays in cultured cells of transgenic rodents constitute, in many ways, feasible “replacements” for in vivo mutagenicity experiments in the corresponding animals [2]. These in vitro models offer significant advantages over their counterparts in vivo animal models as they are much less costly and laborious, require far less time to be completed and, most importantly, do not involve direct use of the animals [3,4,5]. At the same time, the in vitro models have inherent shortcomings that may limit their representation of the in vivo events occurring in animal models (see the Discussion). Nevertheless, if used properly and in well-designed experiments, the in vitro models can provide an initial indication of the mutagenic potential of a given chemical/agent(s), and the results can be used as a guide to devise ‘refined’ in vivo experiments with a “reduced” number of animals [2,4].

The Big Blue^®^ rodent cell culture model and the λ Select *cII* Mutation Detection System (Stratagene Corp., La Jolla, CA, USA, acquired by Agilent Technologies Inc., Santa Clara, CA, USA, BioReliance/Sigma-Aldrich Corp., Darmstadt, Germany) have been successfully employed for mutagenicity testing of carcinogens by many research groups throughout the world [2,4,6,7,8,9,10,11,12]. For the past 16 years, our laboratory has investigated the mutagenic effects of a wide range of chemical and/or physical carcinogens using cultures of the Big Blue^®^ mouse embryonic fibroblasts and the λ Select *cII* assay [13,14,15,16,17,18,19,20,21,22,23,24,25,26,27]. The genome of transgenic Big Blue^®^ mouse houses multiple copies of a λLIZ shuttle vector containing two mutational reporter genes, namely the *cII* and *lacI* transgenes, that are integrated, at a single locus, into chromosome 4 in a head-to-tail arrangement [2,4,6,28]. The λ Select *cII* assay is based on the retrieval of the λLIZ shuttle vectors from the genomic DNA of Big Blue^®^ mouse cells treated in vitro with a test compound, followed by packaging of the rescued vectors into λ phage heads capable of infecting an indictor *Escherichia coli* (*E. coli*). The infected bacteria are then grown under selective conditions to allow for scoring and analysis of mutations in the *cII* transgene [2,6].

Here, we review techniques and principles involved in the preparation and culturing of transgenic Big Blue^®^ mouse embryonic fibroblasts, treatment in vitro with chemical and/or physical agent(s) of interest, determination of the *cII* mutant frequency by the λ Select *cII* assay and establishment of the mutation spectrum by DNA sequencing. We outline various approaches for data analysis and interpretation of the results. We showcase representative studies in which the Big Blue^®^ mouse cell culture model and the λ Select *cII* Mutation Detection System have been used for mutagenicity testing of various chemical and/or physical carcinogens. Lastly, we highlight the advantages of this approach and discuss its limitations, while outlining auxiliary methods, where applicable. We note that the focus of this review is the λ Select *cII* mutation assay and its applications in transgenic Big Blue^®^ mouse embryonic fibroblasts for in vitro mutagenicity testing. Comparing and contrasting the wide variety of existing transgenic mutation assays are beyond the scope of the present review, and we refer interested readers to comprehensive and exhaustive reviews on this and other related topics (see, for example [4,29]).

## 2. Materials and Methods

### 2.1. Generation of Big Blue^®^ Mouse Embryonic Fibroblasts and Preparation of Cell Cultures

Primary mouse embryonic fibroblasts are isolated from embryos derived from Big Blue^®^ mouse on C57BL/6 or B6C3F1 genetic backgrounds, according to our published protocol with some modifications [30]. Briefly, mouse embryos are harvested in utero at 13.5 days of gestation. Following the removal of the head (for DNA genotyping) and internal organs, individual embryos are placed in a centrifuge tube and incubated with collagenase in a serum-free culture medium (e.g., Dulbecco’s Modified Eagle’s Medium (DMEM)) (0.1% *v*/*v*) for up to four hours at 37 °C. At hourly intervals, the embryo-collagenase solutions are gently centrifuged, and the pellet is washed with serum-free media and then re-incubated with the collagenase solution for another hour. Upon completion of digestion, the collected cells are transferred to 150-mm petri dishes containing DMEM, supplemented with 10% fetal calf serum (FBS) and non-essential amino acids, and subsequently cultured at 37 °C. Following 3–4 days of cultivation, confluent cultures are harvested, and cells are snap frozen (i.e., 2–3 × 10^6^ cells per vial). Upon thawing, early passage mouse embryonic fibroblast cultures (*p* < 3) can be established and used for experimental purposes. For mutagenicity experiments, monolayer cell cultures (~50–70% confluent) at a density of ~1 × 10^6^ cells per 150-mm petri dish are prepared in triplicate for each experimental and control condition.

We note that although not as inexpensive as trypsin, collagenase possesses several superior features for embryo digestion. It is not inactivated by divalent cations and can be used with culture medium, with the result that all dissociated cells are provided with nutrients during the digestion. Furthermore, the substrate of collagenase is collagen, an extracellular matrix protein, whereas trypsin is a nonspecific protease that can seriously damage cell membrane proteins, such as hormone and other receptors [31]. Collagenase digestion produces more viable cells, which are less stressed, and have an increased in vitro lifespan compared to their trypsin-digested counterparts.

### 2.2. Cytotoxicity Examination of Test Chemical/Agent

Prior to conducting a mutagenicity experiment, cytotoxicity examination of the test chemical/agent must be performed. This is to establish a biologically-relevant dose range in which the test compound may exert its (potential) genotoxic effects, without causing excessive cell death. The latter is of importance because induced DNA damage by a test compound can only be translated into mutation in viable and proliferative cells. Miscoding DNA lesions must evade repair and undergo translesion DNA synthesis during DNA replication, thereby potentially causing mutation [2]. For cytotoxicity examination, Big Blue^®^ mouse embryonic fibroblasts (approximately 0.5–1 × 10^4^ cells) are seeded onto each well of a multi-well plate (12- or 24-wells). Following overnight incubation, the cells are washed thoroughly with a buffer solution (e.g., phosphate-buffered saline (PBS)) at least 3 times. The test compound, dissolved in an appropriate solvent (e.g., dimethylsulfoxide (DMSO)), is then mixed with cell culture medium at increasing concentrations in a series of screw-cap centrifuge tubes. Dilution mixtures of the test compound and culture medium are then added to each well of the multiwall plate in triplicate. The concentrations of the test compound in a serial dilution may range from mid-nanomolar to low millimolar, with the dilution factor being usually 5 or 10. As the negative control, cell culture medium containing the highest concentration of the used solvent is tested under the same experimental conditions. The duration of treatment varies, depending on test chemical/agent, but usually does not exceed 24 h, considering the finite proliferative capacity of the cells. On average, primary Big Blue^®^ mouse embryonic fibroblasts undergo cell division every 28–30 h for up to 5–7 rounds. Following the treatment, cells are washed multiple times with an appropriate buffer solution and subsequently harvested for evaluation of cytotoxicity using cell proliferation assays, e.g., relative cell counts (RCC), relative increase in cell counts (RICC) and relative population doubling (RPD), or the trypan blue dye exclusion technique or colorimetric tests, such as the MTT assay [32]. The latter two assays, however, may underestimate cytotoxicity as the trypan blue dye exclusion assay detects only cells that die of necrosis, and the MTT assay does not detect cells that eventually die from apoptosis [33]. The cytotoxicity data are used to establish a dose-response survival curve from which biologically-relevant doses of the test compound can be inferred for mutagenicity experiments. For any given test chemical/agent, at least a “low” dose and a “high” dose, resulting in high and low cell survival, respectively, are chosen for mutagenicity testing. Additional “in-between” doses may also be included to establish the existence of dose-response mutagenic effects. For further information on the cytotoxicity examination and selection of concentrations for test compounds, readers are referred to the OECD Genetic Toxicology Guidelines [34,35,36].

### 2.3. In Vitro Treatment for Mutagenicity Testing

Early passage Big Blue^®^ mouse embryonic fibroblasts are treated in vitro with chemical and/or physical agent(s) of interest in comparison to the control. Following the treatment, cells are washed multiple times with an appropriate buffer solution and subsequently grown in complete growth medium for 5–8 days, with cultures being passed when they reach ~80% confluency. Upon completion of the culturing period, cells are harvested, pelleted by centrifugation and stored at −80 °C until analysis. At the time of harvesting, cells are expected to have undergone 3–4 population doublings, a requisite for the fixation of mutations into the genome [2]. 

### 2.4. Genomic DNA Isolation

High molecular weight genomic DNA from treated cells and controls is isolated using standard phenol:chloroform extraction methods or various commercially available DNA isolation kits (e.g., DNeasy Blood & Tissue Kit, Qiagen, Valencia, CA, USA) [5]. The DNA is dissolved in TE buffer (10 mM Tris-HCl, 1 mM EDTA, pH 7.5) and kept at −80 °C until further analysis.

### 2.5. The λ Select cII Assay

**Figure 1:**
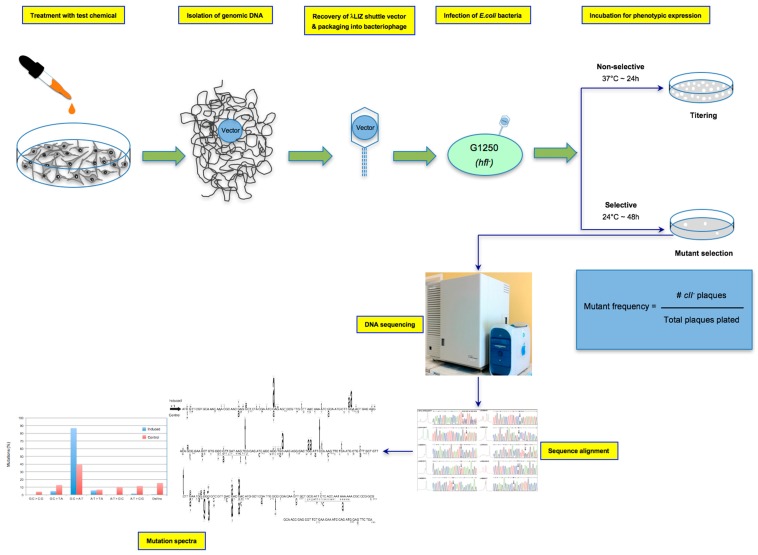
Schematic presentation of the λ Select *cII* assay. The assay is based on the retrieval of the λLIZ shuttle vectors, containing the *cII* transgene as a mutational reporter gene, from genomic DNA of Big Blue^®^ mouse cells treated in vitro with test chemical/agent(s) relative to the control, packaging the vectors into λ phage heads capable of infecting an indicator *E. coli* and performing temperature-sensitive incubation of the bacterial culture to phenotypically express the mutated *cII* transgene [2,4,5,6,28]. Determination of the induced *cII* mutant frequency and establishment of the mutation spectrum by DNA sequencing are highlighted.

The λ Select *cII* assay is used for the detection of mutations in the *cII* transgene recovered from the genomic DNA of cells derived from Big Blue^®^ rodents [2]. The genome of transgenic Big Blue^®^ rodents contains multiple tandem copies of the chromosomally-integrated λLIZ shuttle vector, which carries the *cII* and *lacI* transgenes, as mutational reporter genes [4,6,28]. The λ Select *cII* assay is based on the retrieval of the coliphage vectors from genomic DNA, followed by phenotypic expression using a temperature-sensitive bacterial assay [6]. Briefly, the recovered vector is packaged into viable bacteriophages, and the infective λ phage particles are introduced into an appropriate strain of *E. coli*. Both in-house prepared or commercially-available λ packaging extracts can be used for packaging reactions. The commonly-used Transpack Packaging Extract kit (Stratagene Corp., La Jolla, CA, USA, acquired by Agilent Technologies Inc., Santa Clara, CA, USA, BioReliance/Sigma-Aldrich Corp., Darmstadt, Germany) [37] consists of red tubes containing the first packaging reaction mix (~10 μL), blue tubes containing the second packaging reaction mix (~70 μL for, at least, five reactions) and G1250 *E. coli* as the indictor bacteria. Detailed instructions for the recovery of the λ shuttle vectors, packaging into bacteriophages and infection of the host *E. coli* are provided in all commercial reagent kits [37]. The λ phages multiply lysogenically or lytically in the host *E. coli*, depending on the status of *cII* transcription [38]. The cII protein is required for activation of the *cI* repressor and lambda integrase, both of which are essential for lysogenization [38]. Whereas wildtype cII protein triggers lysogenic cycles in the host *E. coli*, mutated cII protein leads to lysis of the infected bacteria, which manifests as plaques formed on the bacterial lawn [5,6]. The λLIZ shuttle vector also harbors a *cI857* temperature-sensitive (*ts*) mutation that makes the cI (ts) protein labile at temperatures exceeding 32 °C [6]. At temperatures greater than 32 °C, therefore, all vector-bearing phages, regardless of the *cII* mutation status, replicate through lytic cycles in the indicator *E. coli* [4,6]. This temperature sensitivity is the basis for the *cII* selection system in which incubation of the phage-infected bacteria under selective conditions (i.e., 24 °C for ~48 h) results in phenotypic expression of the *cII* mutants, thus forming plaques in a special agar plate [6]. Under non-selective incubation conditions (i.e., 37 °C for ~24 h), both wildtype and mutant cII are indiscriminately expressed, resulting in the formation of plaques in the agar plate [6]. The ratio of plaques formed under the selective conditions to those formed under the non-selective conditions is conventionally referred to as the “*cII* mutant frequency” [4,5]. A schematic presentation of the λ Select *cII* assay is shown in Figure 1.

### 2.6. The Induced and Spontaneous cII Mutant Frequencies and Mutation Spectra

Genomic DNA of Big Blue^®^ mouse embryonic fibroblasts treated in vitro with test chemical/agent(s) and appropriate control is isolated, and retrieval of the λLIZ shuttle vectors and packaging into bacteriophages are achieved using in-house prepared or commercially available packaging extracts. As described in the preceding section (see Section 2.5), after pre-adsorption of the λ phages to G1250 *E. coli*, the bacterial culture is grown on TB1 agar plates under “selective conditions: incubation at 24 °C for ~48 h” (for screening of mutations in the *cII* transgene), as well as under “non-selective conditions: incubation at 37 °C for ~24 h/overnight” for titering (i.e., calculating the total number of mutant and non-mutant plaques screened in the genomic DNA). The *cII* mutant frequency is calculated by dividing the number of plaques formed under the selective conditions to the total number of plaques formed under the non-selective conditions (see Figure 1). For quality control, commercial packaging reaction kits provide standard phage solutions that contain a mixture of wildtype and mutant *cII* with known mutant frequency. The control phage solutions should be included in all assay runs [37]. Furthermore, it is recommended that a minimum of 3 × 10^5^ rescued phages be screened for each experimental and control condition [37]. Depending on the strength of the test compound to induce mutation, 50–150 verified *cII*-mutant plaques are usually sequenced for mutation spectra analysis, with the stronger mutagens requiring fewer numbers of plaque for DNA sequencing (see below).

For mutation spectra analysis, putative *cII* mutant plaques are selected at random and re-plated at low density under selective conditions to verify the mutant phenotype and isolate single plaques for DNA sequencing. The re-plating of putatively mutated plaques serves two purposes: (a) plating artifacts may sometimes be mistaken for small-size plaques; and (b) an agarose core taken from a screening plate may contain non-mutant phage(s) together with a mutant phage. Secondary plaques from a low-density re-plating will provide an uncontaminated mutant template for polymerase chain reaction (PCR) and subsequent DNA sequence analysis. Following the re-plating, individual well-isolated plaques are picked, transferred to a microcentrifuge tube containing 25 μL double-distilled water, boiled for 5 min and centrifuged at maximum speed for 3 min. Ten microliters of the supernatant are immediately transferred to a new microcentrifuge tube containing 40 μL of a PCR mastermix in which the final concentrations of the reagents are 1× *Taq* PCR buffer, 10 pmol each of the forward and reverse primers, 12.5 nmol of each dNTP and 2.5 U of *Taq2000* DNA Polymerase (Qiagen, Valencia, CA, USA). The forward and reverse primers include 5′-CCACACCTATGGTGTATG-3′ (positions -68 – -50) and 5′-CCTCTGCCGAAGTTGAGTAT-3′ (positions -345 – -365), respectively. A 432-bp product containing the *cII* gene and flanking regions is amplified by PCR using the following cycling parameters: a 3-min denaturation at 95 °C, followed by 30 cycles of 30 s at 95 °C, 1 min at 60 °C and 1 min at 72 °C, with a final extension of 10 min at 72 °C. The PCR amplified product is purified using the QIAquick PCR purification kit (Qiagen, Chatsworth, CA, USA) and sequenced using various DNA sequencing platforms. The resulting DNA sequences are analyzed by alignment with the reference *cII* sequence using software programs, such as the web-based SeqWeb or T-Coffee sequence alignment servers.

## 3. Data Analysis

### 3.1. Determining the Mutagenic Potency of a Test Chemical/Agent and Analyzing the Sequence-Specificity of Mutations

**Figure 2:**
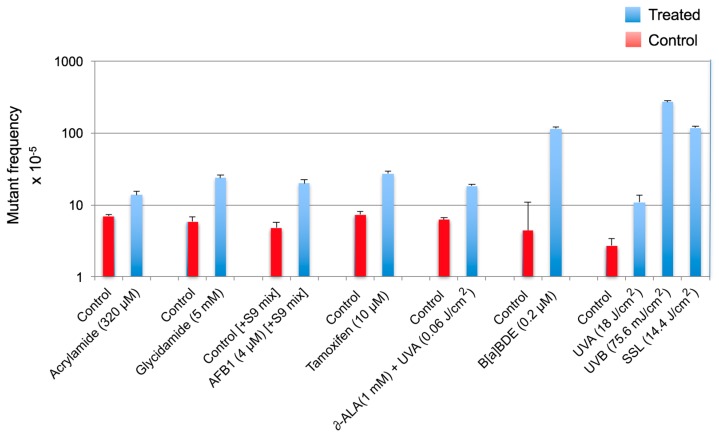
Comparison of the *cII* mutant frequencies induced by various chemicals and/or physical agents in Big Blue^®^ mouse embryonic fibroblasts. Data are from our published studies on acrylamide [15], glycidamide [17], aflatoxin B1 (AFB1) [25], tamoxifen [21] and δ-aminolevulinic acid (δ-ALA) plus low dose ultraviolet light A (UVA: λ > 320–400 nm) [18], benzo(a)pyrene diol epoxide (B(a)PDE) [22] and equilethal doses of UVA, UVB (λ = 280–320 nm) and simulated sunlight UV (SSL) [24]. To efficiently metabolize aflatoxin B1 in Big Blue^®^ mouse cells, the S9-activation system (S9 mix) consisted of Aroclor 1254-induced rat liver preparations and cofactor reagents was used [25].

**Figure 3:**
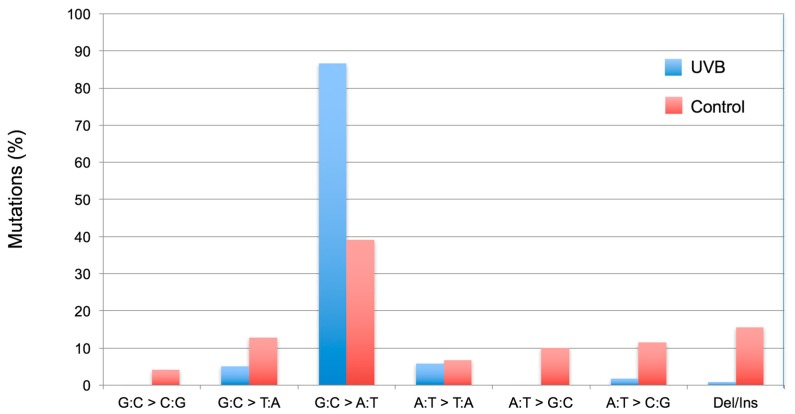
Induced *cII* mutation spectrum in Big Blue^®^ mouse embryonic fibroblasts irradiated with UVB relative to the control. Data are from our published study [26]. The strand mirror counterparts of all transitions (e.g., G → A and C → T) and transversions (e.g., G → T and C → A or G → C and C → G) are combined. Ins = insertion; Del = deletion. The UVB-induced mutation spectrum is characterized by significant increases in the relative frequency of single or tandem C → T transitions at pyrimidine dinucleotides.

**Figure 4:**
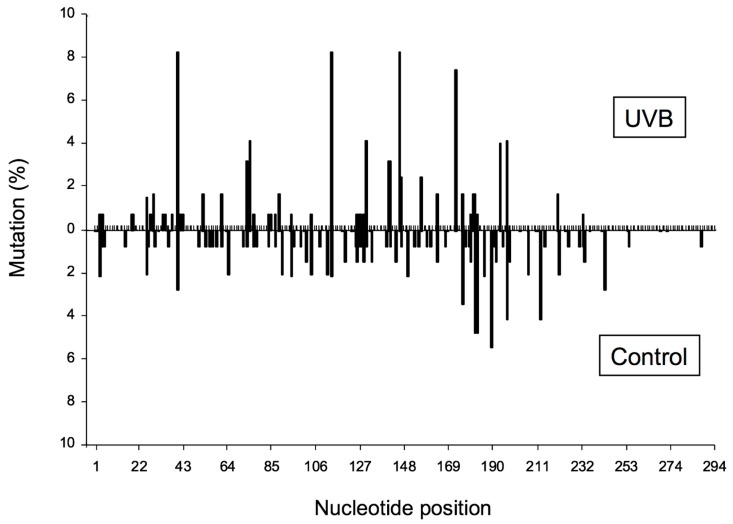
Bar chart presentation of the induced and spontaneous *cII* mutation spectra in Big Blue^®^ mouse embryonic fibroblasts irradiated with UVB and the control, respectively. Data are from our published study [26]. The location and percentage of mutations along the *cII* transgene in UVB-irradiated cells and control are indicated by bars above and below the reference sequence, respectively.

**Figure 5:**
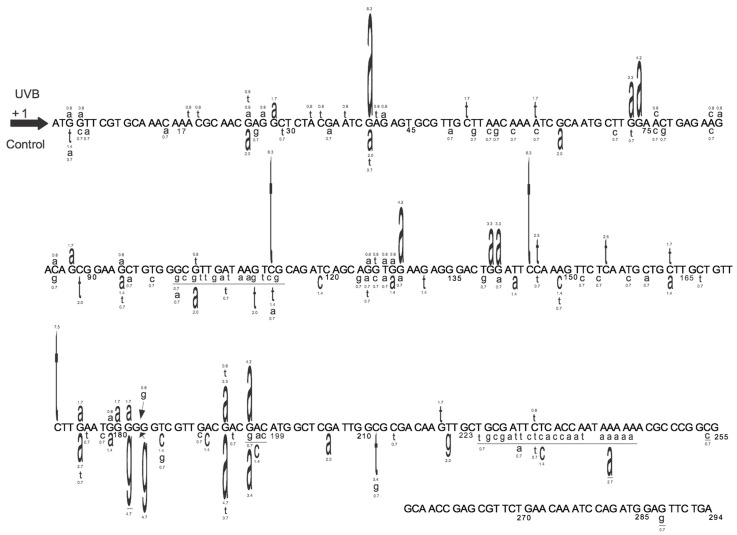
Detailed map of the induced and spontaneous *cII* mutation spectra in Big Blue^®^ mouse embryonic fibroblasts irradiated with UVB and the control, respectively. Data are from our published study [26]. The UVB-induced *cII* mutations are typed above the reference sequence, whereas the spontaneous mutations (control) are typed below the reference sequence. The height of a mutated base represents its frequency of mutations (i.e., the higher the base, the more frequently mutated). Numbers above a mutated base indicate its percentage frequency of mutations. Deleted bases are underlined. Inserted bases are shown with an arrow. Numbers below the bases are reference nucleotide positions.

The extent of increase in relative *cII* mutant frequency, which is characterized by elevation of the *cII* mutant frequency in the genomic DNA of cells treated with a test compound relative to the control (e.g., solvent-treated cells), represents the strength of the tested compound to induce mutation. The fold-increase in *cII* mutant frequency may vary from a few to several hundred, depending on the test compound (see Figure 2). “Sequence-specificity” of mutations induced by the tested compound can be determined by DNA sequencing of the *cII*-mutant plaques recovered from the genomic DNA of cells treated with the examined chemical/agent as compared to the control. Graphical representations of the respective DNA sequencing data are commonly referred to as the “induced” and “spontaneous’ mutation spectra, respectively. The induced and spontaneous mutation spectra are visualized in different formats (see Figure 3, Figure 4 and Figure 5). All presentations are graphic forms of comparing and contrasting the distribution and frequency of various types of mutation (e.g., base substitution, insertion or deletion) in the *cII* transgene of mutant plaques recovered from the genomic DNA of treated cells versus control. For example, Figure 3 is a histogram presentation of the induced and control mutation spectra, showing the overall percentage of each type of mutation (e.g., G → A transition or G → T transversion) in the *cII* transgene in treated versus control samples. A bar chart display of the induced and control mutation spectra, highlighting the location and frequency distribution of mutations along the *cII* transgene in treated versus control samples, is shown in Figure 4. Figure 5 is a detailed map of the induced and control mutation spectra, in which the type, location and frequency distribution of mutations in the *cII* transgene in treated versus control samples are shown. Determination of the locations and site-specific frequency of mutations in the *cII* transgene induced by a test compound relative to the control is known as ‘mutation spectra analysis’. Comparative analysis of the induced and spontaneous (control) mutation spectra provides information on the sequence-specificity of mutations induced by the test compound.

### 3.2. Statistical Analysis and Interpretation of the Results

(I) Mutant frequency: Depending on the data distribution, parametric or non-parametric tests are used to determine the significance of difference in the *cII* mutant frequency between treatment and control groups (i.e., induced versus spontaneous mutant frequencies). Comparison of the induced *cII* mutant frequencies across different treatment groups is made by various (pairwise) statistical tests, as applicable. Examples of various statistical tests that can be used to compare differences in mutant frequency between a treatment and control group or across different treatment groups are provided in our published studies, in which acrylamide [15], glycidamide [17], aflatoxin B1 (AFB1) [25], tamoxifen [21], δ-aminolevulinic acid (δ-ALA) plus low dose ultraviolet light A (UVA: λ > 320–400 nm) [18], benzo(a)pyrene diol epoxide (B(a)PDE) [22] and equilethal doses of UVA, UVB (λ = 280–320 nm) and simulated sunlight UV (SSL) [24] were tested for mutagenicity (see Figure 2). For further information on the comparison of mutant frequencies, readers are referred to the OECD guidance regarding pairwise significance testing, trend test and comparison of the responses to the range of historical negative controls [34,35,36]. A positive has a significantly increased response, a positive trend and at least one response that exceeds the historical range of negative controls. If only one or two are true, the response is equivocal, and more testing may be called for [34,35,36].

(II) Mutation spectra: The hypergeometric test of Adams and Skopek is commonly used to compare the overall induced and spontaneous mutation spectra [39], although other tests, such as the χ^2^ test or analysis of variance (ANOVA), can also be used to compare the frequency of each specific type of mutation (e.g., transition, transversion, insertion or deletion) between the induced and control mutation spectra, or among various mutation spectra induced by different chemicals/agents or by various doses of the same chemical/agent. For example, in the corresponding study for Figure 3 [26], the χ^2^ test was used to compare the overall percentage of each type of mutation (e.g., transitions, transversions or insertion/deletion) in the *cII* transgene in UVB-irradiated versus control cells. Furthermore, the hypergeometric test of Adams and Skopek was employed to compare the location and frequency distribution of mutations along the *cII* transgene in the UVB-irradiated versus control cells, as shown in both Figure 4 and Figure 5 [26].

## 4. Discussion

Transgenic rodent cell culture models are invaluable supplements to the corresponding animal models for mutagenicity testing of carcinogens. In addition to circumventing the direct use of animals, these in vitro cell culture models enable mutation analysis at a fraction of the cost, time and labor as those required for the in vivo experiments in animal models [3,4,5]. The in vitro cell culture models, however, may not fully represent all aspects of mutagenesis in vivo. For instance, absent metabolic capacity or reduced proficiency of cultured cells to convert certain chemicals into DNA-reactive species might not reflect DNA-damage driven mutagenicity in animals exposed in vivo to genotoxic chemicals [2,4]. To offset this drawback, an external metabolic activation system (i.e., S9 mix) can be added to the in vitro cell culture models [40]. This axillary approach, however, may not be straightforward because exogenous metabolic activation mixtures prepared from different species may differently simulate mammalian metabolism (e.g., rat versus human-derived S9 mix). In addition, introduction of an external metabolic activation system to a cell culture model precludes tissue-specific or cell-type-dependent biotransformation, which is a determinant of the mutagenicity of many carcinogens [41,42].

Moreover, the route of exposure and delivery of carcinogens to animals, which are highly complex and often organ-specific, require modification to become compatible with the in vitro cell culture models [43,44]. For example, chemicals whose route of exposure is inhalation (e.g., tobacco smoke) are often made amenable to in vitro testing after they are converted from gaseous or vapor forms to liquid or condensate. This change in chemical properties is likely to complicate the pharmacokinetics and pharmacodynamics of the tested compound, however. Furthermore, the finite lifespan of normal cells in culture does not permit modeling of chronic exposure to carcinogens in vivo [2,4]. Both rat and mouse transgenic cell lines with virtually indefinite proliferative capacity are available for long-term experiments [8,10,11,45], although the extent to which immortalized cell lines accurately replicate primary cells remains a matter of debate [46]. Admittedly, mimicking real life human exposure to genotoxic chemicals/agents is more challenging in in vitro cell culture models than in experimental animals [47,48,49]. Nonetheless, mutagenicity testing in the former models can provide initial data that may prove instrumental in designing in vivo studies in appropriate animal models [2,4].

Elegant and comprehensive reviews have discussed the advantages and disadvantages of analyzing mutations in transgenes as compared to endogenous genes [4,29]. The weight of evidence suggests that transgenes and endogenous genes respond relatively similarly to genotoxic chemicals when direct comparisons have been made [24,25,50,51,52,53,54,55,56], although important differences may also exist [13,57,58,59,60]. For instance, spontaneous mutant frequency tends to be several-fold higher in transgenes than in endogenous genes; however, fold-increases in induced mutant frequency in transgenes are usually greater than those in endogenous genes (reviewed in [4]). For the most part, however, these two differences cancel each other out, resulting in similar (if not higher) “sensitivity” for mutation detection in transgenes relative to endogenous genes [4]. Furthermore, transgenes seem to be transcriptionally inactive, as evidenced by the absence of corresponding mRNA in various tissues of transgenic animals (reviewed in [4,29]). This is important because transcription-coupled repair (TCR), which preferentially removes DNA lesions from the transcribed strand of endogenous genes, is a significant determinant of mutagenesis in experimental animals and humans alike [61,62]. The reduced DNA repair capacity of transgenes due to the absence of TCR, however, may be advantageous for the detection of induced mutations. This is due to the fact that transgenes with diminished DNA repair proficiency are more likely to accumulate DNA lesions and mutations than endogenous genes. This together with the high copy number of transgenes relative to single copy endogenous genes may contribute to the high sensitivity of transgenes for mutation detection.

Transgenes have high CG content and are heavily methylated [4], which may impact DNA-damage formation, as many genotoxic chemicals/agents are known to preferentially bind to methylated CpG dinucleotides in the genome [63,64,65]. In addition, whereas selection plays an important role in mutagenesis in endogenous genes, transgenes appear to be neutral [50,66,67,68]. This is supported by the observation that induced mutant frequency in highly proliferating tissues of transgenic animals remains persistently elevated long after termination of exposure to carcinogens [29]. The induced mutant frequency in tissues from which mutagenized cells transit as part of their development (e.g., bone marrow), however, may not remain as persistent [29].

There are constraints on types of recoverable mutation in transgenes. For example, the overwhelming majority of mutations recovered from analysis of the *cII* transgene in the Big Blue^®^ system are point mutations, involving base substitutions and less frequently small frameshifts (insertion/deletion) [4,69]. Large deletions that extend into or through sequences necessary for λ phage propagation would not be detected as they will not be recoverable [6,69]. Furthermore, there are size limitations for in vitro packaging, requiring λ vectors to have *cos* sites separated by 38–51 kb. Therefore, insertions and deletions that produce vectors outside this size range will not be recovered in the Big Blue^®^ system [50,69,70,71]. The *gpt delta* ^(*Spi−*)^ and *LacZ* plasmid-based mouse systems are available for the detection and characterization of such large deletion mutations, however [4,72]. 

In the Big Blue^®^ system, the λ Select *cII* assay serves as a positive selection test since cells containing the wildtype phages are selected against before the mutations arise [6]. As such, ex vivo mutations occurring in the bacteria infected with lesion-bearing phages or in vitro mutations arising spontaneously during growth of the phages in bacteria are unlikely to be recognized/detected in this assay [4,6,73]. This is a significant “specificity” advantage for the assay because the above artefactual mutations are not of mammalian origin and do not represent a response to the DNA damage induced by the test chemical/agent(s). Likewise, the “sensitivity” of the assay for the detection of mutations induced by various test chemicals/agents has proven to be reproducibly high. Of relevance for transgenic cell culture models, prolonged in vitro culturing of cells may lead to accumulation of oxidative stress-induced DNA damage [74,75,76,77], which manifests as elevation of spontaneous mutant frequency a few-fold higher than that found in vivo in tissues/organs of the corresponding transgenic animals [4]. Altogether, although for a select number of chemicals or agents that have been tested both in cultured cells of transgenic mice/rats and the corresponding animals treated with the test compound, mostly consistent results have been observed, disparate findings have also been reported [4,29].

## 5. Conclusions

In summary, the Big Blue^®^ rodent cell culture model and the λ Select *cII* assay constitute a versatile approach for mutagenicity testing of carcinogens. The possibility of preparing primary cell cultures from various tissues/organs of transgenic Big Blue^®^ animals, the feasibility of treating cultured cells in vitro with virtually any test chemical/agent, the easy recovery of the λ shuttle vector carrying a relatively short reporter gene amenable to rapid DNA sequencing (*cII*; 294 bp versus *lacI*: 1080 bp, *LacZ*: 3021 bp, *gpt*: 456 bp [4]) make this approach highly desirable for initial testing of suspect mutagens. More recently, we have expanded the applications of this approach by developing a novel technique in which a revised version of the λ Select *cII* assay coupled to next-generation sequencing platforms offers high throughput analysis of mutations in a time-, cost- and labor-effective manner [26].

## Abbreviations

ANOVA	analysis of variance
DMEM	Dulbecco’s Modified Eagle’s Medium
DMSO	dimethylsulfoxide
*E. coli*	*Escherichia coli*
FBS	fetal calf serum
PBS	phosphate-buffered saline
